# COVID-19 orphans—Global patterns associated with the hidden pandemic

**DOI:** 10.1371/journal.pgph.0000317

**Published:** 2022-08-31

**Authors:** Callum Lowe, Leli Rachmawati, Alice Richardson, Matthew Kelly

**Affiliations:** 1 Department of Global Health, National Centre for Epidemiology and Population Health, Australian National University, Canberra, Australia; 2 Centre for Indonesia’s Strategic Development Initiatives, Central Jakarta, Indonesia; 3 Statistical Support Network, Australian National University, Canberra, Australia; Pontificia Universidad Javeriana, COLOMBIA

## Abstract

Whilst the COVID-19 pandemic has caused significant mortality across the globe, many children have been orphaned due to the loss of their parents. Using the framework of an ecological analysis, we used estimates of total maternal/paternal orphans using an online COVID-19 orphanhood calculator to estimate the total orphans per COVID-19 death for 139 countries. Descriptive statistics were used to determine global patterns behind this risk of children being orphaned. Linear regression models were fitted to determine factors associated with this risk, and the association with vaccination coverage was calculated. We found that there is tremendous global variation in the risk that COVID-19 deaths will lead to orphaned children, and that this risk is higher in countries below median GDP per capita (1·56 orphans per deaths) compared to countries above (0·09 orphans per death). Poverty prevalence (B = 2·32, p<0·01), GDP per capita (B = -0·23, p<0·05), and a greater proportion of people with NCDs being reproductive aged (B = 1·46, p<0·0001) were associated with this risk. There was a negative correlation between 2^nd^ dose vaccination coverage and orphans per death (p<0·05). The risk of children being orphaned per COVID-19 death, alongside fertility rate, is due to there being a greater share of COVID-19 deaths among younger persons. This is more likely in poorer countries and those where the age distribution for non-communicable diseases that elevate COVID-19 mortality risk are more uniform. Due to vaccine coverage inequity, more children will suffer the loss of their parents in poorer countries.

## Introduction

The COVID-19 pandemic has resulted in health, economic and social crises around the world, causing significant morbidity and mortality. As of December 10, there were 5,285,888 deaths from COVID-19 worldwide [1]. Most COVID-19 deaths are among adults and the elderly. Smith et al (2021) estimated the case fatality rate for children <18 years old in England to be two per million, [2] compared to almost one in four in Italy [3]. Particularly with the advent of the Omicron variant, coronavirus is extremely infectious, and can spread quickly to all members of a household. Coupled with the low risk of mortality among children, there is great possibility that children will survive COVID-19 infection while their parents or caregivers will not. As such, there exists a unique risk for COVID-19 orphans, the ‘hidden’ pandemic.

A modelling study by Hillis et al (2021) was the first to quantify the burden of orphans due to the COVID-19 pandemic [4]. The study took mortality and fertility rates by age and sex in 21 countries to model estimates of the number of children affected by the pandemic, either through the loss of one or both parents, caregivers, or custodial grandparents. The findings estimate that between March 1, 2020 and April 30, 2021, 1,134,000 children experienced one of the above deaths. This is roughly a third of the 3,322,107 deaths as of April 30 due to COVID-19, indicating the immense scale of this challenge [5]. Whilst little academic research has pertained to the issue of COVID-19 orphans, the issue has been documented in different countries globally, such as Peru [6] and Indonesia [7]. Field-interviews have uncovered the drastic consequences this experience can have on individuals and families, such as the reports of women needing to take care of as many as eight children due to parental death [6].

Whilst little research has investigated the distribution and effect of orphans due to the COVID-19 pandemic, this phenomenon has been observed in other pandemics. The 2014 Ebola Virus Disease outbreak in West Africa led to an estimated 9,600 orphans [8]. Indeed, orphanhood can have extreme long-term impacts on children’s lives, both in terms of physical and mental health [9]. Racial disparities exist in the impact of orphanhood in midlife [10]. Losing one or both parents can be extremely difficult for children; orphans might receive less love and attention and are more prone to behavioural and emotional problems [10]. These challenges will ultimately have an impact on social life and mental health. Orphans can experience problems with home life, classroom learning, and recreational activities, and are also susceptible to child labor demands [11]. An additional challenge from the COVID-19 pandemic is the shift to distance learning, which in turn will exacerbate the negative impact of being orphaned due to limited social interaction. Orphans experience a greater risk of disease and malnutrition [12] and sexual abuse, [13] and have higher rates of depression, posttraumatic stress disorder, suicidal thoughts, and anxiety [14]. These issues are bolstered in low- and middle-income countries, where social support services for orphaned children are both fewer and less extensive than high-income countries [15]. This is a unique risk in the COVID-19 pandemic, because coronavirus transmission is high on nearly every continent.

The findings of Hillis et al (2021) however suggest major variation in the burden of orphanhood worldwide after holding the number of COVID-19 deaths constant [4]. The study estimates the rate of orphaned children is 2·02 per death in Angola, compared with 0·13 in Australia–a 15-fold difference [4]. This indicates that the risk of a child being orphaned per death in Angola is 15 times higher than in Australia. Whilst fertility rate is a significant factor, influencing the number of children at risk, the difference in fertility rates between Angola (5·4) and Australia (1·7) for example [16] is insufficient to explain the 15-fold difference in the risk of children being orphaned per death. It may be possible that differences between countries such as poverty rate and the proportion of people with co-morbidities that are current parents of children are also responsible for this discrepancy.

Understanding the patterns of discrepancies between countries will shine light on the scarcely discussed issue of COVID-orphans in existing literature. Directing greater attention towards this issue will encourage an enhanced global effort to support and prevent the burden of further orphans. As such, we aim to address this gap in the literature by extending the work of Hillis et al [4] to investigate the patterns and factors associated with the risk of COVID-19 orphans globally.

## Methods

To determine patterns associated with, and the distribution of the risk of children being orphaned due to the COVID-19 pandemic, we conducted an ecological analysis at a global level. An ecological analysis was suitable as we aimed to look at global variation between countries. The basis of our analysis is that of the work of Hillis et al (2021), whereby estimates for the total number of children orphaned due to the COVID-19 pandemic were calculated [4]. This study estimated the total orphans caused by excess mortality during the COVID-19 pandemic using age and sex disaggregated mortality and fertility rates for 21 countries. Following this, a linear model with a log link for the number of orphans (caused by maternal/paternal, primary caregiver, and grandparent mortality) was fitted, and day-to-day estimates for orphans of each category in all countries was made available online [17]. For the purposes of our analysis, we only considered maternal/paternal orphans which comprise one of the most detrimental categories of orphanhood.

### Calculating orphans per death

To obtain estimates of the total number of maternal/paternal orphans for all countries, we used the online COVID-19 orphanhood calculator, developed as part of the study by Hillis et al (2021) to extract the estimated number of maternal/paternal orphans up to September 28, 2021 [4]. The details of this calculation are available in the supplementary material section of Hillis et al (2021) [4]. Briefly, maternal/paternal orphans were estimated by first estimating the number of children each adult of different age/sex groups would have in 2020. This was then aggregated into 5-year age bands and multiplied by the excess mortality rate to estimate total maternal/paternal orphans. The authors then adjusted for ‘double’ (maternal and paternal) orphans to obtain a reliable estimate. This calculation was performed for 21 countries and then extrapolated in the online COVID-19 orphanhood calculator using a logistic model relying on the high correlation between orphans per death and total fertility rate [4]. We then used this estimate of the total number of maternal/paternal orphans and then divided by the total cumulative number of COVID-19 deaths up to the same date in each country obtained from the Johns Hopkins Coronavirus Resource Center [18]. By dividing the estimated number of maternal/paternal orphans by the total number of COVID-19 deaths, we obtain the dependent variable used in analysis termed orphans per death (OPD). As the extrapolated estimates of the number of orphans for the countries not included in the study of Hillis et al (2021) were based on COVID-19 deaths only and not excess mortality, [4] we divided total estimated orphans by COVID-19 deaths only and not excess deaths.

### Calculating covariates

As we aimed to also look at potential variables that correlate with OPD, we collected country-level covariates based on the hypothesis that where a greater proportion of a countries’ deaths are among persons of reproductive age (i.e. more likely to be parents of children), then the risk of children being orphaned is greater. We conducted a literature search for co-morbidities that might elevate the risk of COVID-19 mortality among people of reproductive age. Non-communicable diseases (NCDs) identified were hypertensive heart disease (HHD), diabetes, obesity, and cardiovascular disease [19, 20]. We hypothesised that these conditions, alongside indicators of country level socio-economic status might correlate with OPD. The way in which NCDs might correlate with OPD is that in some countries, sufferers of these NCDs may be generally younger than in other countries and therefore more likely to be at the age of a child’s parent. We also included HIV and stroke; the former might correlate with OPD as HIV would be expected to elevate the risk of death (from any condition), and the latter likely correlates with the aforementioned NCDs.

We collected data on NCD prevalence using the Global Burden of Disease (GBD) Results Tool using data for 2019 estimates [21]. NCD prevalence was calculated as the proportion of all persons with the disease in a given country/province that were aged 15–49 years (reproductive age), as opposed to the prevalence in that age group. This decision was made because if such conditions were associated with greater risk of COVID-19 mortality, then it would be expected that in countries/provinces where a greater proportion of persons that suffer that particular condition are reproductive aged then the percentage of all COVID-19 mortality from persons in that age group would also increase. The following equation describes this calculation:

$${NCD}_{i}=\frac{Number\;of\;persons\;with\;{NCD}_{i}\;aged\;15-49}{Total\;number\;of\;persons\;with\;{NCD}_{i}}$$

where i = HHD, diabetes, chronic kidney disease, HIV, stroke.

Thus, we assume that reproductive age (15–49 years) is a good proxy for the age of the majority of parents at the time of the pandemic. For obesity, data was not available on the breakdown of obesity prevalence by age group for each country in the GBD results. Instead, we used the crude obesity prevalence for each country using data from the Global Health Observatory [22].

Country-level indicators of socio-economic status used comprised the prevalence of poverty (obtained from the world population review [22]) and Gross Domestic Product (GDP) per capita (current $US) obtained from the World Bank [23]. These covariates were chosen because we hypothesize that a greater proportion of COVID-19 deaths attributed to younger persons would be associated with poor socio-economic status. Total fertility rate was obtained from the World Bank [15].

The proportion of each countries’ population that was reproductive aged (15–49 years) might be a potential confounder as younger populations may have more deaths among younger persons more likely to be a parent. As such, we collected this variable by calculating the proportion of the population aged 15–49 using UN World Prospects data [24].

Vaccination rate as of 1st December 2021 was tabulated using data from Our World in Data [25]. We extracted the percentage of each countries’ population that had received at least two doses of a COVID-19 vaccine.

### Statistical analysis

Countries included in analysis were those that had full data on all covariates and the dependent variable (OPD). As fertility rate directly linearly scales the risk of children being orphaned (where fertility rate doubles, the number of orphans per death would double), we computed the OPD divided by fertility rate for regression analysis, termed OPD adjusted. This variable can be thought of as ‘households’ or ‘family units’ affected by COVID-19 pandemic orphanhood; however, the fertility rate variable is a national average and so does not differentiate between individual household sizes.

The OPD values were ranked by country according to WHO region (Africa, Americas, Eastern Mediterranean, European, South-East Asia, and Western Pacific). Following this, countries were categorised as having a GDP per capita as below or above the median GDP per capita of countries in the analysis. Subsequently, the kernel density distribution of OPD was plotted by GDP category. Boxplots were also constructed in the same manner. Following this, simple bivariate linear regression models for all covariates with the OPD adjusted variable were created, and the correlation coefficient and p-value for regression were calculated.

Principal components analysis was conducted to group non-communicable disease variables with high multicollinearity into components. The component eigenvalues were plotted on a scree plot and the component explaining the most variance was extracted and scores created using the regression method. The Kaiser-Meyer-Olkin (KMO) test and Bartlett’s Sphericity Test (BST) were computed to assess the suitability of the principal component analysis [26]. A single component was extracted representing the NCD variables. A linear regression model was then fitted to identify significant predictors of adjusted OPD. A logit link for OPD adjusted was used. Dividing OPD by fertility rate acted as a means of controlling for the direct and non-differentiating effect of fertility rate on OPD, and led to a variable with values between 0 and 1. This then meant that the logit transformation could be computed, leading to an outcome on the range (-∞, ∞). A forward selection process was used to identify important variables based on the change in model R^2^ adjusted and visual inspection of residuals. Next, pairwise interactions were assessed for inclusion in a similar way. Finally, the correlation between vaccination rate and OPD was computed in a separate analysis.

Statistical analysis was performed in RStudio 3.5.1 [27].

## Results

**Fig 1:**
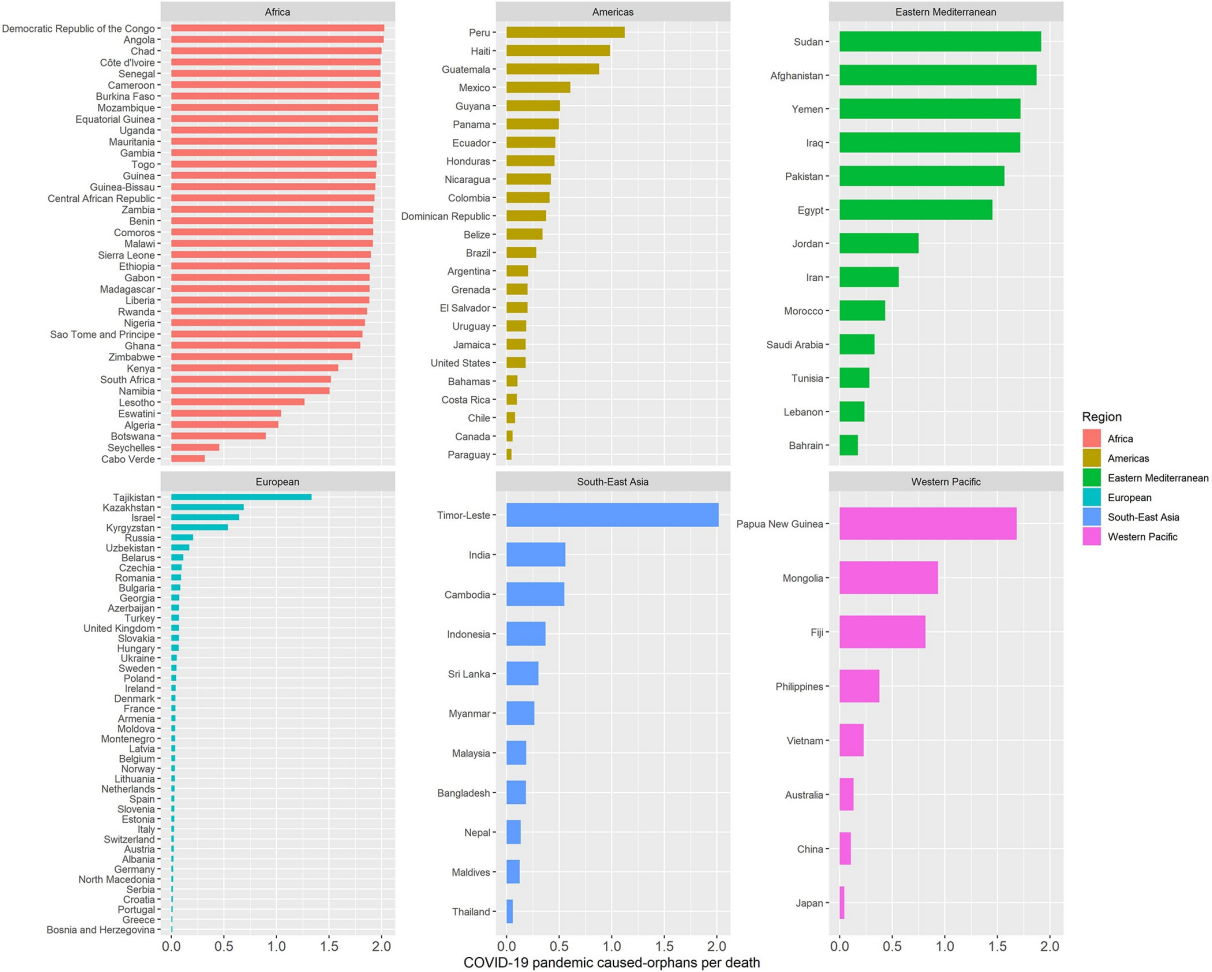
Distribution of orphans per death by country. Countries organized by WHO region.

A total of 139 countries had available data on all covariates and were included in this analysis. In Fig 1 we present the estimates of OPD in these countries by WHO region. The highest OPD rates were observed in Africa ranging between 1·5 and 2 except for 6 countries with values below 1·5. Large variation was observed in the South-East Asia region, with values close to 2 in Timor-Leste, but below 0.5 in all other countries. OPD rates were consistently below 0·25 in European countries except for Kyrgystan, Israel, Kazakhstan and Tajikistan, and varied widely in the Americas; the highest observed in Peru. Variation in Eastern Mediterranean countries was large; the highest was nearly 2 in Sudan and below 0·25 in Bahrain.

**Fig 2:**
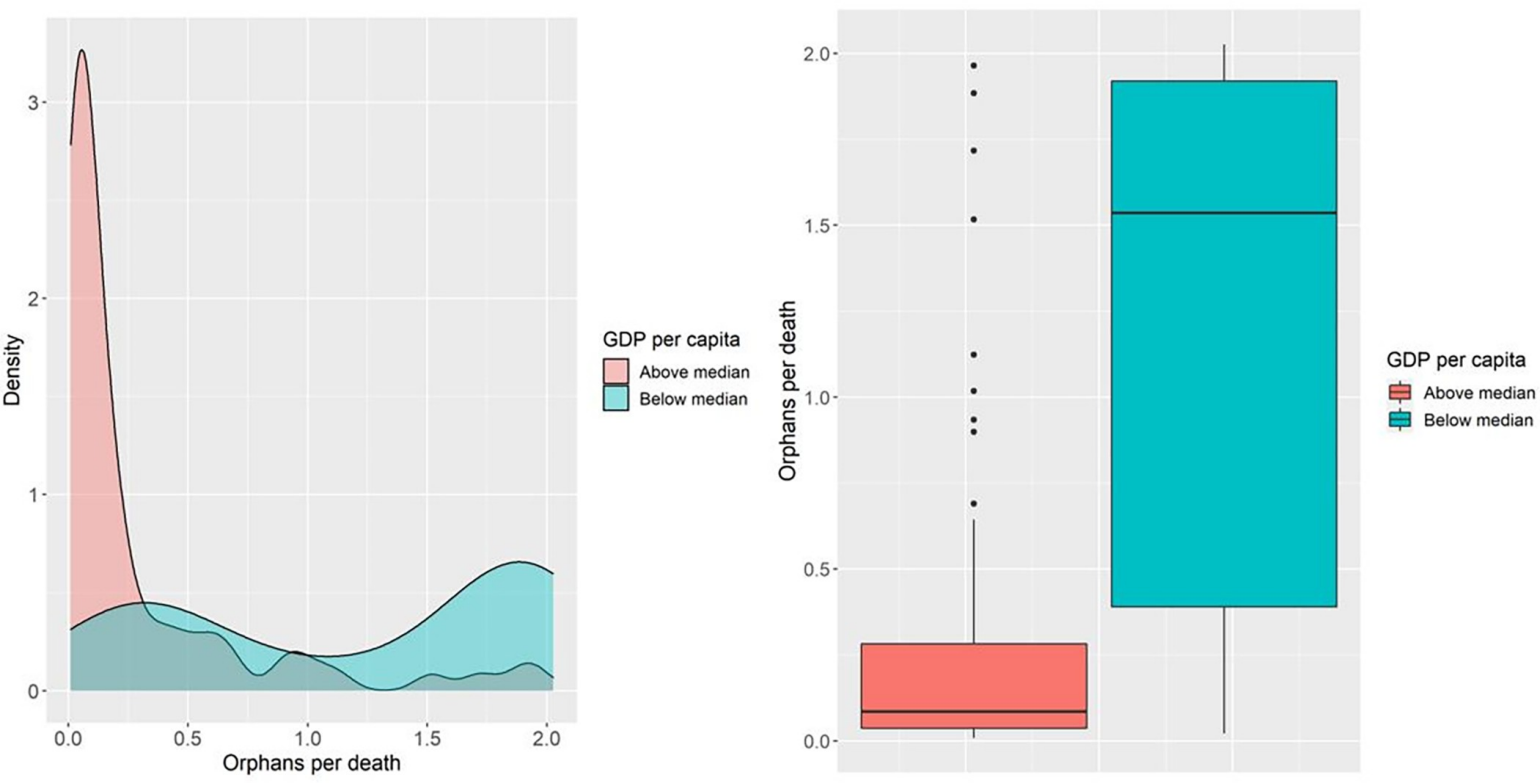
Kernel density functions for OPD by GDP category (Left) and boxplots of OPD by GDP category (right). GDP categories calculated as countries with a GDP below the median value in the study data, and those countries with a GDP above the median value. Median value = US $12,939 per capita.

In Fig 2 we present the kernel density function of OPD (left) and boxplot of OPD (right) stratified by GDP per capita category. Countries with a GDP per capita above the median were right-skewed with the majority having OPD values less than 0·5, whilst countries with a GDP per capita below median had a bimodal density distribution. As a result, the variation in higher income countries was extremely small in comparison to lower income countries, although a number of outlier values were present. The median OPD value for lower income countries (1·56) was 17 times greater than for higher income countries (0·09).

**Fig 3:**
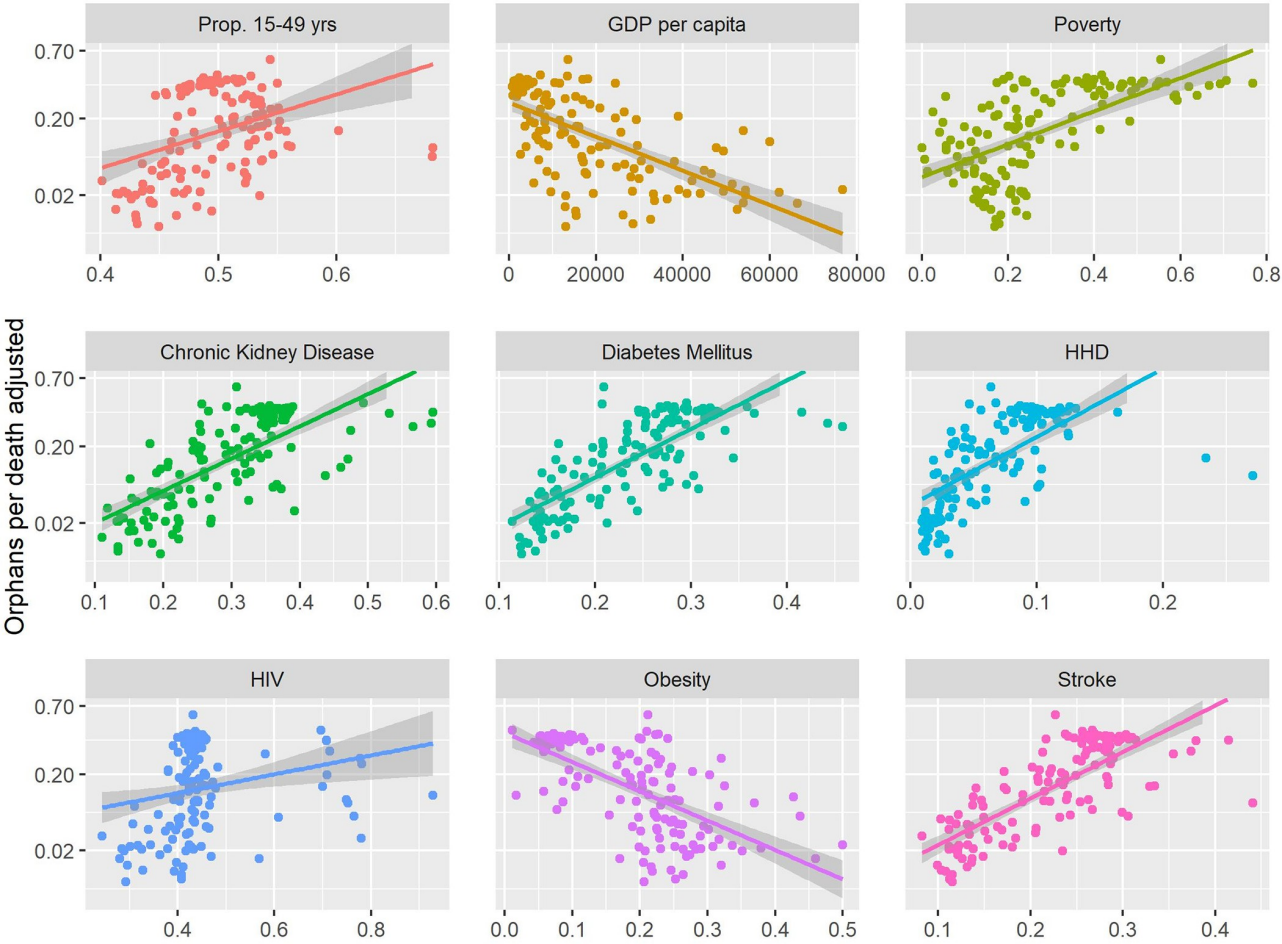
Simple univariate linear regression models for the correlation between covariates and OPD Adjusted (OPD divided by fertility rate). x-axis expressed as proportion (between 0 and 1) except for GDP per capita (expressed as $US). P-values calculated for regression model. Y-axis transformed to the logit scale. Note that the y-axis intervals are not evenly spaced. Orphans per death adjusted = orphans per death divided by national average total fertility rate. Solid lines represent regression prediction, grey bands represent 95% confidence interval. Chronic Kidney Disease, Diabetes Mellitus, HHD, HIV, and stroke measured as proportion of country population with condition that are aged 15–49 years. Obesity and poverty measured as proportion of population. GDP per capita measured as $US.

Scatter plots with univariate linear regressions for OPD adjusted on each covariate are shown in Fig 3. Statistically significant associations were positive for % population reproductive aged (R = 0·21, p<0·.05), poverty (R = 0·70, p<0·0001), and the proportion of chronic kidney disease (R = 0·66, p<0·.0001), diabetes (R = 0·71, p<0·0001), HHD (R = 0·61, p<0.0001) and stroke (R = 0·70, p<0·0001) cases among people of reproductive age. The associations with obesity and GDP per capita were negative and statistically significant (R = -0·66, p<0·0001 and R = -0·62, p<0·0001 respectively).

**Fig 4:**
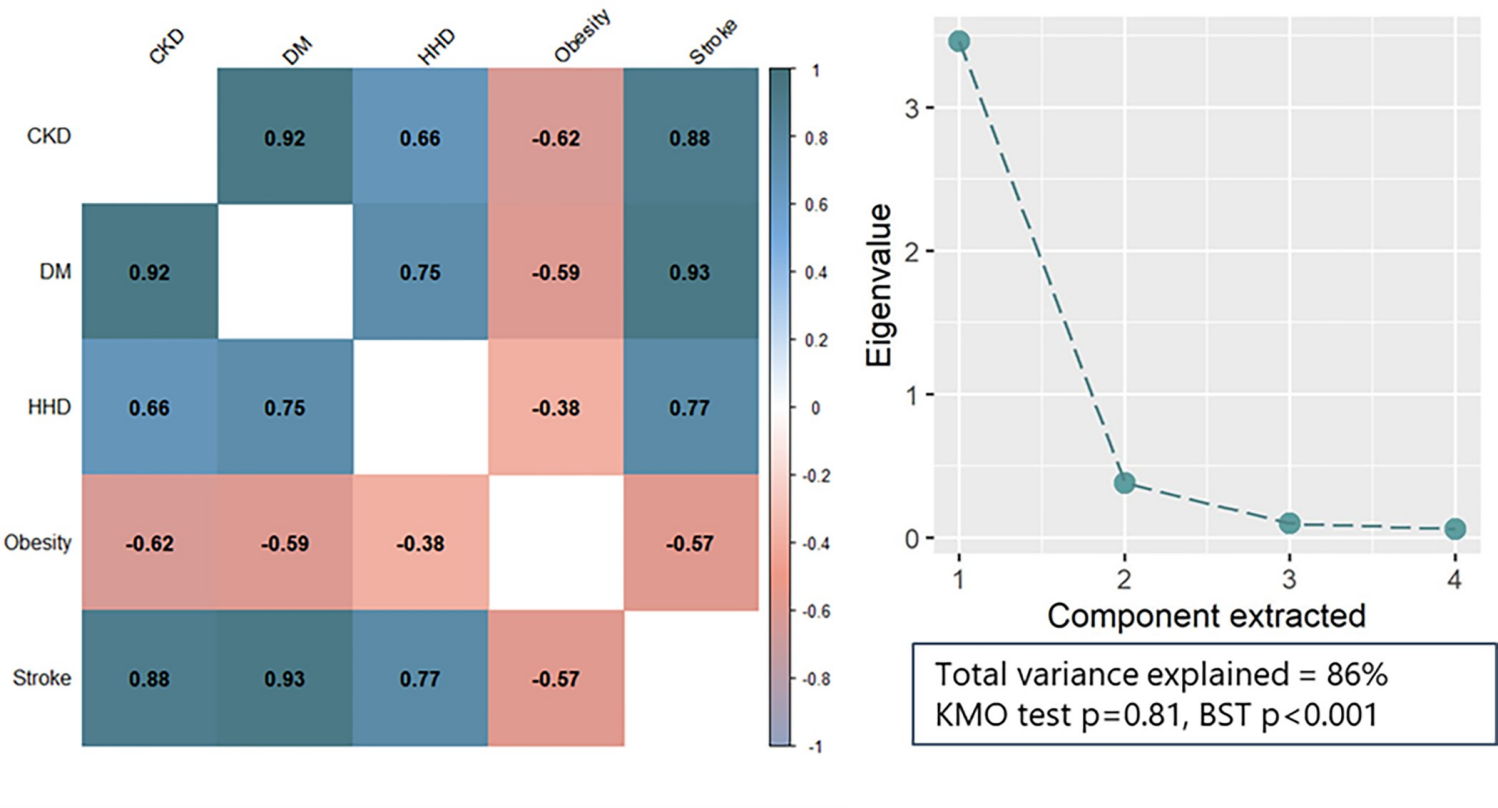
Correlation matrix for covariates tabulated in the analysis (left) and principal components analysis results (right). Scree plot (right) for total variance explained for included variables in principal components analysis (CKD, Diabetes, Stroke, HHD). CKD, Chronic Kidney Disease. DM, Diabetes mellitus. HHD, Hypertensive Heart Disease, HIV, Human Immunodeficiency Virus. KMO, Kaiser-Meyer-Olkin test. BST, Bartlett’s Sphericity Test.

The correlation matrix (left) and principal components analysis (right) for NCD variables is shown in Fig 4. There was generally strong positive correlation between NCD variables except for obesity where the correlation was negative with all other NCD variables. On the basis of this, all NCD variables except obesity were included in principal components analysis. The scree plot (right) indicated that the majority of variation (86%) in the four entered NCD variables could be explained in a single component. This component can be thought of as a general variable indicative of greater proportion of NCDs occurring among younger people.

Following principal components analysis, the first component was extracted and component scores were extracted using the regression method to compute the NCD principal component score variable. Subsequently, multiple linear regression models were fitted to OPD adjusted with a logit link (Table 1). NCD component scores, poverty and GDP per capita all had statistically significant associations with OPD adjusted at p<0·0001 in univariate regressions. In the multiple regression model, these associations remained significant; NCD component score (β = 1·46, 95% CI = 0·95, 1·97, p<0·0001), poverty prevalence (β = 2·32, 95% CI = 0·65, 3·99, p<0.01), and GDP per capita (β = -0·23, 95% CI = -0·41, -0·05, p<0·05). The NCD component score was the only covariate to have an increased coefficient in the multiple regression model whilst poverty and GDP per capita coefficients reduced in magnitude.

**Table 1:** Multiple linear regression model for OPD adjusted with a logit link function.

**Covariates (univariate models)**	**Estimate (SE)**	**95% CI**	**p**
NCD Principal component score	1·18 (0·08)	1·02, 1·34	**<0**·**0001**
Poverty	5.41 (0.58)	4·27, 6·55	**<0**·**0001**
GDP per capita	-0.56 (0·06)	-0·68, -0·44	**<0**·**0001**
**Covariates (multiple model)**	**Estimate (SE)**	**95% CI**	**p**
NCD Principal component score	1·46 (0·26)	0·95, 1·97	**<0**·**0001**
Poverty	2·32 (0·85)	0·65, 3·99	**<0**·**01**
GDP per capita	-0·23 (0·09)	-0·41, -0·05	**<0**·**05**
**Interaction terms**			
NCD×Poverty	-1·54 (0·65)	-2·81, -0·27	**<0**·**05**
NCD×GDP	-0·12 (0·06)	-0·27, 0·00	**<0**·**05**
Poverty×GDP	0·21 (0·48)	-0·73, 1·15	0·67

Model fit: R = 0·84, R^2^ = 0·71, Adjusted R^2^ = 0·69, p<0·001

NCD, Non-communicable disease. GDP, Gross Domestic Product. Poverty covariate measured as prevalence as percentage of total population. GDP per capita calculated in units of U.S Dollars x 10,000. Covariates in the multiple model were adjusted for the percentage of population reproductive age. OPD adjusted = OPD divided by national average fertility rate. Statistically significant associations have p-values in bold typeface.

**Fig 5:**
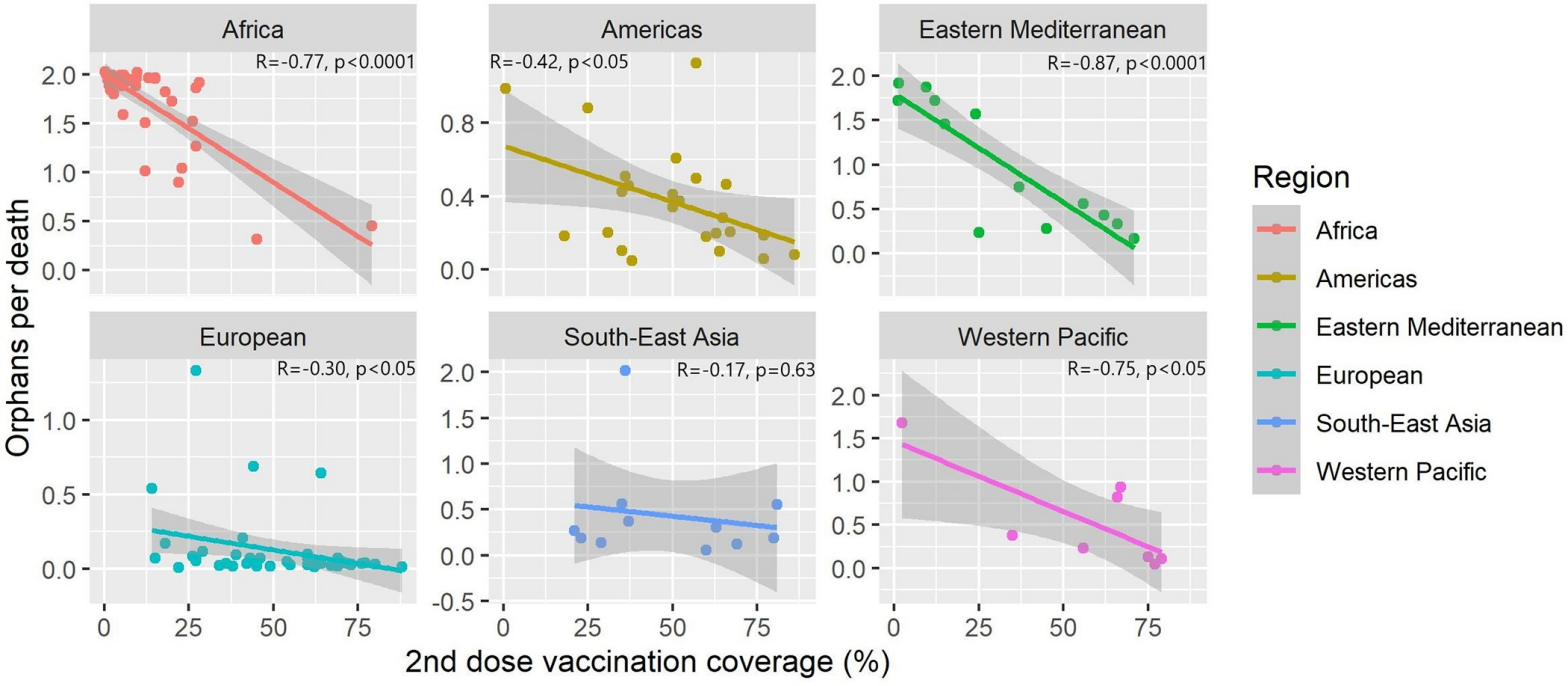
Association between 2^nd^ dose vaccination coverage and OPD by region. Correlation coefficient and p-value for univariate linear regression models displayed in text in main panels. Gray bands represent 95% confidence intervals. Vaccination data as of 1^st^ December 2021.

Finally, univariate associations between 2^nd^ dose COVID-19 vaccination coverage and OPD by region are presented in Fig 5. There is a statistically significant negative association between vaccination coverage and OPD in all regions except for South-East Asia (R = -0·17, p = 0·63). This association was strongest in Eastern Mediterranean countries (R = -0·87, p<0·0001), Africa (R = -0·77, p<0·0001) and also Western Pacific (R = -0·75, p<0·05). The association was weaker in the Americas (R = -0·42, p<0·05). There was strong clustering in African countries with extreme high values of OPD and low values of vaccination coverage. European countries had significant clustering of OPD values below 0·25, despite large variation in 2^nd^ dose vaccination coverage.

## Discussion

The COVID-19 pandemic so far has caused significant mortality around the world, but has also led to a ‘hidden pandemic’ of children orphaned by the loss of one or both parents. In this ecological analysis, we investigated the distributions of the risk of children being orphaned from COVID-19 deaths and found that this risk is significantly higher in lower income countries and those with greater prevalence of poverty. Furthermore, we found that in countries where a greater proportion of people suffering comorbidities associated with elevated COVID-19 mortality risk are reproductive aged, the risk of children being orphaned per death was greater.

A higher risk of children being orphaned per COVID-19 death is intrinsically linked to the age distribution of COVID-19 mortality. Flatter distributions will mean that a greater share of deaths are among younger persons which will lead to the death of more parents. Where the distribution is right skewed and deaths are predominantly among the elderly, few deaths among parents of young children will occur, and so the risk of children being orphaned per death is diminished.

We found that there was a statistically significant association between OPD and poverty prevalence as well as GDP per capita (Fig 3, Table 1). This result is consistent with the observation that COVID-19 age-mortality distributions are flatter among poorer countries [28]. One possible explanation for this association is that in poorer countries, access to healthcare and treatment is more limited than in richer counterparts. The number of hospital beds (per 100,000 population) is correlated with GDP, and therefore in poorer countries fewer hospital beds would be expected to correlate with more COVID-19 deaths across all age groups [28]. Thus, the proportion of deaths attributable to people of reproductive age would rise, and as such the risk of children being orphaned per death would also rise.

We also found that when a greater proportion of a countries’ population that suffer particular NCDs are reproductive aged there was a positive association with OPD (Fig 3, Table 1). This can be explained by the notion that when a greater share of diseases that elevate COVID-19 mortality risk are among persons of parenting age, then a greater share of COVID-19 deaths will be among this group whose death is more likely to lead to a child being orphaned. These findings are supported by the work of Nepomuceno et al (2020) who suggested that flatter age-mortality distributions in low- and middle-income countries are due to a greater proportion of chronic diseases among younger persons in these countries [29]. We found this to be true for hypertensive heart disease, chronic kidney disease, diabetes, and stroke (Fig 3). We did not find this association for obesity, however this may be due to tabulating obesity as overall national prevalence and not the proportion of obese people aged 15–49. We also found in post-hoc analysis that obesity prevalence correlated strongly with GDP, and therefore may be confounded subject to confounding.

It is a particular concern that the impact on children being orphaned due to the pandemic is likely to be greater in low and middle income countries, because the capacity of governments to manage orphaned children and facilitate social support might be lower than in higher income countries [9]. Orphaned children may face ongoing challenges with nutrition, schooling, financial support and psychosocial support [30].

These issues are further exacerbated by the fact that countries with a greater OPD rate tended to have a lower vaccination coverage. In countries where vaccination coverage is lower, COVID-19 associated mortality rates will be higher, and therefore the subsequent numbers of children orphaned will follow in a similar fashion. In more developed countries however, not only is the vaccination rate higher, but the risk of children being orphaned is also lower. Therefore, of the fewer deaths that will occur in these more developed countries, the risk that those deaths will lead to children being orphaned is reduced. These issues underscore the tragedy that is unequal distribution of COVID-19 vaccines across the globe. Our results highlight the need for vaccine equity across the globe. While many have argued for vaccine equity from the perspective of preventing mortality and reducing the risk of potentially more severe coronavirus variants, our results show that in addition to this, the prevention of children being orphaned will also be tied directly to even vaccination coverage.

This study is not without limitations. Our analysis relied on estimates of orphans produced from an extrapolation that was only based on 21 countries [4]. The extrapolation was only based off of fertility rate and total COVID-19 deaths and not each countries underlying mortality and fertility rate distribution. As such, we have regressed data that is not obtained empirically which therefore may lead to reduced reliability in the estimates of our associations. However, the extrapolation based off fertility rate in the aforementioned study relied on a high correlation (R^2^ = 0·93). As such, we recommend that the numerical estimates produced from our study are carefully interpreted, and large variation between countries is more valid than comparison of similar countries. Furthermore, we could not assess obesity in the units of the proportion of persons with obesity aged between 15–49 years, and so by reverting to country obesity prevalence we may not have captured this association as accurately as the other NCD variables. Under- or over-estimation of COVID-19 deaths in each country and variability in the systems to track mortality will directly affect the estimate of orphans per death, and as such our results are subject to variation due to the reporting of COVID-19 deaths. We only considered orphans due to the loss of parents in this analysis, and not from the loss of grandparents or other caregivers. However, our aim was to look at patterns associated with arguably the most severe form of orphanhood–the loss of a child’s parents. Future research could study whether the same variations occur when including these categories. In line with the ecological study framework, we were not able to capture differences between people within countries. Whilst we identified the risk of OPD to be higher in poorer countries, within many countries there is likely inequity in the risk of OPD whereby poorer communities within the same country are likely to be burdened by more COVID-19 orphans. We did not take into account excess mortality in the calculation of OPD as the extrapolated estimates of orphans did not consider excess mortality. Thus, there may exist other factors predicting the risk of children being orphaned due to non-COVID-19 deaths during the pandemic.

Our study serves as a reminder of the perpetual cycle of poverty experienced in poorer countries, furthered by the elevated risk of children being orphaned per COVID-19 death. Our findings underscore the need for uniform vaccination coverage across the globe, which will minimize the number of deaths among all demographics including parents, and therefore minimize the number of children becoming orphaned. Our findings also suggest that the proportion of persons with NCDs that are aged 15–49 years also correlates with OPD independently of poverty and GDP.
